# Prognostic Value of Different Levels of Uric Acid in Patients with Coronary Chronic Total Occlusion Undergoing Percutaneous Coronary Intervention

**DOI:** 10.3390/jcm12113794

**Published:** 2023-05-31

**Authors:** Mohamed Ayoub, Kambis Mashayekhi, Michael Behnes, Tobias Schupp, Muharrem Akin, Jan Forner, Ibrahim Akin, Franz-Josef Neumann, Dirk Westermann, Volker Rudolph, Aurel Toma

**Affiliations:** 1 Division of Cardiology and Angiology, Heart Center University of Bochum, 32545 Bad Oeynhausen, Germany; 2 Department of Internal Medicine and Cardiology, Mediclin Heart Centre Lahr, 77933 Lahr, Germany; 3 Department of Cardiology, Angiology, Haemostaseology and Medical Intensive Care, University Medical Centre Mannheim, Medical Faculty Mannheim, 68167 Mannheim, Germany; 4 Department of Cardiology and Angiology, Hannover Medical School, 30625 Hannover, Germany; 5 Department of Cardiology and Angiology II, University Heart Center Freiburg, 79189 Bad Krozingen, Germany; 6 Department of Internal Medicine II, Division of Cardiology, Medical University of Vienna, 1080 Vienna, Austria

**Keywords:** chronic total occlusion, percutaneous coronary intervention, uric acid, hyperuricemia, mortality, coronary artery disease

## Abstract

Recent data suggest that uric acid (UA) might be an independent predictor of clinical outcomes following percutaneous coronary intervention (PCI). The predictive value of uric acid in patients undergoing PCI for chronic total occlusions (CTO) is unknown. We included patients with CTO who underwent PCI at our center in 2005 and 2012, with available uric acid levels before angiography. Subjects were divided into groups according to uric acid tertiles (<5.5 mg/dL, 5.6–6.9 mg/dL, and >7.0 mg/dL), and outcomes were compared among the groups. Out of the 1963 patients (mean age 65.2 ± 11 years), 34.7% (*n* = 682) had uric acid concentrations in the first tertile, 34.3% (*n* = 673) in the second tertile, and 31% (*n* = 608) in the third tertile. Median follow-up was 3.0 years. Uric acid levels in the first tertile were associated with significantly lower all-cause mortality, as compared to the third tertile, with an adjusted hazard ratio (HR) of 0.67 (95% confidence interval (CI): 0.49 to 0.92; *p* = 0.012). No significant differences regarding all-cause mortality were found between patients in the first and second tertiles (HR: 0.96 [95% CI: 0.71 to 1.3; *p* = 0.78]). High levels of uric acid emerged as an independent predictor of all-cause mortality in patients with chronic total occlusion treated with PCI. Hence, uric acid levels should be incorporated into the risk assessment of patients with CTO.

## 1. Introduction

Coronary artery disease (CAD) is one of the leading causes of death worldwide. With 607,000 deaths, CAD was the single most common cause of death in US American adults in 2005 [1]. Advances in the treatment of CAD have improved patient outcomes and prognosis during recent years. Detailed assessment by coronary angiography provides the basis for individually well-matched revascularization therapies, including percutaneous coronary intervention (PCI) with drug-eluting stents (DESs) or coronary artery bypass grafts (CABG) [1].

Chronic total occlusions (CTO) are common angiographic findings in patients with CAD, and are detected in 15–30% of patients who undergo cardiac catheterization [2,3]. By definition, a CTO is a 100% occlusion in the coronary artery with thrombolysis in myocardial infarction (TIMI) and a grade flow of 0 (true CTO). This occlusion has to be present for a duration of at least three months [4]. Revascularization with PCI in such patients may be challenging, mainly due to firm, calcified CTO lesions and rather complex coronary anatomy. In such cases, it is often only possible to choose retrograde access, which is associated with a higher peri-interventional risk [5]. Given the increased procedural complexity, lower success rates of about 70% are achieved in CTO PCI, as compared to 96% in non-CTO PCI [2]. However, especially in patients with comorbidities, who are at high risk of CABG or who are even inoperable, CTO PCI may be the method of choice [6].

Uric acid (UA) is the final product of purine metabolism and is metabolized by xanthine oxidase. UA has been shown to be related to comorbidities, including arterial hypertension, type 2 diabetes, metabolic syndrome, and chronic kidney disease [7]. Moreover, it is currently debated if UA can be classified as an independent risk factor for CAD and/or mortality [8]. This makes the evaluation of UA levels a promising tool for the assessment of cardiovascular risk and mortality in high-risk patient populations.

Here, we investigate the relation between UA levels and all-cause mortality in a large series of patients with coronary CTOs. We hypothesized that CTO patients with higher uric acid levels have an increased mortality risk, as compared to patients with lower or normal levels of uric acid.

## 2. Materials and Methods

### 2.1. Study Population and Laboratory Parameters

Written informed consent was obtained beforehand and the collection of data was performed in accordance with the Declaration of Helsinki, and approved by the institutional review board. Patients who underwent PCI at our institution between January, 2005 and December, 2012 for coronary CTO were included in this analysis. CTO patients presented with either angina (CCS classification 2–4), exertional dyspnea (NYHA 2–4), or ischemia on non-invasive testing.

CAD was diagnosed in the presence of ≥50% lumen obstruction in one of the three major coronary arteries, and CTO was defined as angiographic evidence of a thrombolysis in myocardial infarction (TIMI) flow grade 0 within an occluded arterial segment for more than 3 months [4]. In the absence of previous angiographic documentation, the duration of occlusion was estimated clinically based on the onset of symptoms or timing of myocardial infarction (MI) in the CTO-related artery. PCI was performed according to the applying standard guidelines, and the success of CTO PCI was angiographically defined as the successful recanalization of the lesion with residual stenosis <30% and TIMI flow grade 3. The attending cardiologist was responsible for concomitant treatment and management of each patient. Before the PCI procedure, data on cardiovascular risk factors and clinical characteristics of all patients were collected and venous blood samples were taken to assess kidney function and measure uric acid, high-density lipoprotein cholesterol (HDL-C), and low-density lipoprotein cholesterol (LDL-C).

### 2.2. Clinical Outcomes and Follow-Up

The primary endpoint was all-cause mortality. A follow-up protocol consisted of prospectively obtained data from hospital readmission, outpatient records, and telephone interviews with the patient and/or referring physician at 30 days, 1 year, and 3 years following PCI.

### 2.3. Statistical Analysis

Baseline and procedural characteristics, as well as lesion characteristics are shown with means and standard deviations or median (interquartile range, IQR) or using the Student *t*-test. Categorical variables were expressed as percentages and were compared using Pearson’s chi-square test or Fisher’s exact test. UA levels were divided into tertiles. Cumulative event rates were calculated and graphically described according to the Kaplan–Meier method. We derived hazard ratios (HR) with associated 95% confidence intervals (CI) from univariable and multivariable Cox proportional hazards models. Follow-up was censored at 3 years. Survival curves were constructed for time-to-event variables with Kaplan–Meier methodology and compared using a log-rank test. The log-rank test was used to compare survival between patients with uric acid levels in the different tertiles. Multivariable analyses were calculated with the Cox regression models for the prediction of 3-year all-cause mortality. Adjusted hazard ratios (HRs) with 95% confidence intervals (CIs) were reported. Known predictive factors, including sex, body mass index (BMI), diabetes, hypertension, dyslipidemia, chronic kidney disease, prior MI, prior CABG, left ventricular (LV)-dysfunction (EF < 40%), successful CTO revascularization, and medication at discharge (ARBs and Allopurinol) were included in the multivariate model. A covariate was allowed in the multivariate model if it influenced the model with a likelihood ratio significance level of *p* < 0.05 and removed if its significance level exceeded *p* > 0.1. All tests were two-sided, and statistical significance was set at 5%. For all statistical analyses, we used the SPSS software package, version 18 (SPSS Inc., Chicago, IL, USA).

## 3. Results

### 3.1. Sample Characteristics

A total of 1963 (16.3%, *n* = 326 women) patients with a mean age of 65 ± 11 years met the inclusion criteria and underwent the CTO PCI procedure. A retrograde approach was used in 23.99% (*n* = 471) patients. The mean numbers of stents implanted per patient were 1.28 ± 0.9 (Tertile 1), 1.3 ± 0.9 (Tertile 2), and 1.3 ± 1.0 (Tertile 3), and total stent lengths (in mm) per patient were 33.7 ± 25.0 (Tertile 1), 34.6 ± 26.2 (Tertile 2), and 36.8 ± 28 (Tertile 3), respectively.

A total of 34.7% (*n* = 682) patients had UA concentrations in the first tertile, 34.3% (*n* = 673) in the second tertile, and 31% (*n* = 608) in the third tertile. Baseline characteristics are summarized in Table 1 (First tertile: <5.5 mg/dL, second tertile: 5.6–6.9 mg/dL, and third tertile: >7.0 mg/dL).

### 3.2. Clinical Outcomes

**Figure 1:**
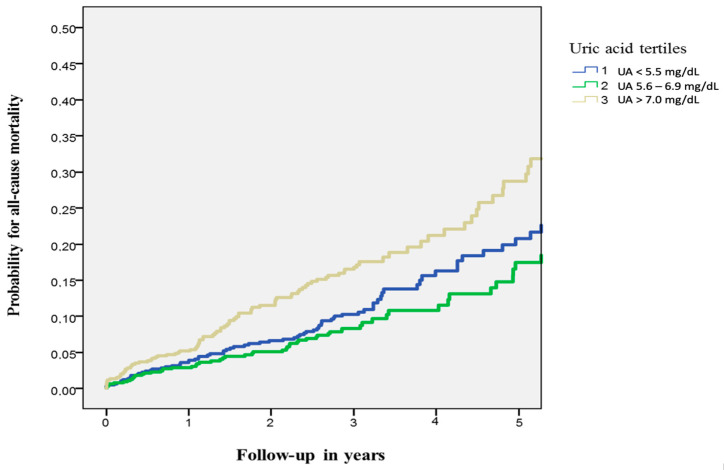
All-cause mortality stratified according to tertiles of uric acid. UA = uric acid level.

CTO PCI was successfully achieved in 83% (*n* = 1630) of patients. Table 2 summarizes procedural characteristics stratified for UA tertiles. All-cause mortality at 5 years was 11.1% in the first tertile, 8.6% in the second tertile, and 16.2% in the third tertile. After multivariable adjustment, all-cause mortality at 5 years was significantly lower in the first tertile compared to the third UA tertile (adjusted HR 0.58, 95% confidence interval [CI] 0.39 to 0.87, *p* = 0.009). No significant differences regarding all-cause mortality were found among patients in the first and second tertiles (adjusted HR: 0.96, 95% CI 0.71 to 1.3, *p* = 0.78, Figure 1). Other independent predictors of all-cause mortality were diabetes, arterial hypertension, chronic kidney disease, and LVEF (Table 3).

## 4. Discussion

This study demonstrated a significant relationship between UA levels and mortality in a large cohort of patients undergoing CTO PCI. Previous studies have shown similar results in patients with acute coronary syndromes (ACS) [10,11] and stable CAD [12,13]. Tscharre et al. investigated a total of 1215 patients with ACS undergoing PCI, followed them for 5.5 years on average, and observed an independent association of UA with long-term major adverse cardiovascular events (MACE) [10]. Ndrepepa et al. enrolled a total of 8149 patients who underwent PCI for stable CAD in their analysis and identified UA as an independent predictor of 1-year mortality [12]. Similar associations in our patient cohort suggest that UA may add to the risk stratification of patients undergoing CTO PCI.

Our results show a significant higher all-cause mortality at 5 years in the third UA tertile (>7.0 mg/dL). This cut-off level is higher compared to values found in previous studies investigating UA and cardiovascular death. Uric acid right for heart health [14], an Italian nationwide, multicenter retrospective, observational cohort study assessed the prognostic cut-off values of serum uric acid (SUA) in predicting fatal and morbid heart failure and found cut-off values of serum uric acid to predict total mortality that were largely within the normal range (4.7 mg/dL, 95% CI 4.3–5.1 mg/dL). Similarly, the cut-off value that better predicted cardiovascular death was within the normal range (5.6 mg/dL, 95% CI 4.99–6.21 mg/dL) [15]. A possible explanation of this discrepancy might be that CTO PCI patients with CTO are significantly more complex and multimorbid.

Pathophysiological mechanisms underlying the relation between UA and adverse events in CAD patients remain to be further elucidated. The role of UA as a possible risk factor for cardiovascular disease and its related comorbidities might serve as an explanation [16]. Uric acid seems to be involved in the development of atherosclerotic plaques [17] and has been shown to enhance atherosclerosis progression by inducing oxidative stress and vascular inflammation [18,19,20]. Both oxidative stress and vascular inflammation are involved in the pathogenesis of restenosis, which can be one of the main reasons for cardiac-related deaths in CTO PCI patients [21]. At the same time, UA is proposed as a risk factor for cardiovascular comorbidities, including the development of arterial hypertension [22], and might play a significant role in the development of diabetes mellitus and metabolic syndrome [7], which are also known risk factors for re-stenosis and cardiovascular death. Uric acid causes activation of the renin–angiotensin system (RAAS) by promoting the production of angiotensin II, a potent vasoconstrictor that increases blood pressure. The activation of the RAAS can also lead to sodium and water retention, which may further increase blood pressure. Furthermore, uric acid has been shown to reduce nitric oxide (NO) bioavailability in the endothelium, which can impair endothelium-dependent vasodilation and increase vascular resistance [23]. In addition, UA seems to be a useful biomarker in the prediction of heart failure, renal dysfunction, left ventricular hypertrophy, as well as stroke and cardiovascular events [24], and might, therefore, serve as a possible biomarker for mortality risk after CTO PCI.

What are the possible clinical implications of the assessment of UA in CTO PCI? Since CTO is often a condition in multimorbid patients, UA might be used as an overall risk marker facilitating clinical decision making and identifying patients in need of continuous and prompt follow-up. In addition, UA might be a directly treatable target in patients who underwent CTO PCI. If UA indeed has an independent pathophysiological role in the progression of cardiovascular disease, lowering UA therapies might, in turn, improve long-term outcomes. A study by Kao et al. demonstrated the beneficial effects of allopurinol on left ventricular mass and endothelial dysfunction in patients with chronic kidney disease [25]. Finally, UA as a routinely assessed serum marker might serve as a useful biomarker in studies investigating CTO treatment. No randomized controlled trial has yet evaluated the clinical outcomes following CTO PCI. However, several studies demonstrated the beneficial effects of successful CTO PCI in terms of sustained improvement in quality of life [26] and improved left ventricular function [27]. In this regard, UA levels could be useful in assessing baseline risk when comparing CTO PCI versus the medical management of CTO in future studies.

Incorporating blood-derived biomarkers into the risk prediction of the outcomes of patients undergoing CTO PCI represents an innovative approach for such a high-risk sub-group of patients. Since biomarkers have been widely incorporated into the risk prediction of patients suffering from heart failure [28,29,30], their additive value in CTO-PCI beyond periprocedural myocardial infarction is widely under-investigated (reference [31,32]).

Our study has a few limitations. The study is limited by its retrospective, single-center design, and UA levels were available at a single point in time. Furthermore, causes of death were not reported. Furthermore, we included patients treated with CTO-PCI in the years 2005–2012. Recent technical advances regarding wires and instruments, including microcatheters, might influence interventional complication rates and mortality.

In conclusion, UA was identified as an independent predictor of mortality in patients undergoing CTO-PCI. Therefore, UA might complement the risk stratification of patients with CTO and identify patients in need of aggressive treatment and close follow-up.

## Figures and Tables

**Table 1:** Baseline characteristics stratified according to uric acid tertiles.

Characteristics	Tertile 1 <5.5 mg/dL (*n* = 682)	Tertile 2 5.6–6.9 mg/dL (*n* = 673)	Tertile 3 >7.0 mg/dL (*n* = 608)	*p*-Value
Age, years, mean ± SD	65 ± 11	65 ± 11	66 ± 11	0.159
Female gender, No. (%)	159 (23.3)	90 (13.4)	77 (12.7)	<0.001
Body mass index, kg/m^2^, mean ± SD	27.3 ± 4.1	28.1 ± 4.0	29.0 ± 4.7	<0.001
Cardiovascular risk factors, No. (%)				
Diabetes mellitus	199 (29.2)	179 (26.6)	207 (34.0)	0.013
Current smoker	140 (20.5)	131 (19.5)	121 (19.9)	0.886
Dyslipidemia	579 (84.9)	585 (86.9)	530 (87.2)	0.418
Hypertension	523 (76.7)	563 (83.7)	536 (88.2)	<0.001
Family history of CAD	264 (38.7)	263 (39.1)	208 (34.2)	0.139
History of myocardial infarction	148 (21.7)	162 (24.1)	176 (28.9)	0.009
Previous PCI	104 (15.2)	104 (15.5)	101 (16.6)	0.773
Previous CABG	84 (12.3)	103 (15.3)	801 (16.6)	0.079
LVEF < 40%	270 (39.6)	246 (36.6)	306 (50.3)	<0.001
Previous failed CTO recanalization	314 (19.0)	56 (16.1)	56 (16.1)	0.22
Laboratory data, mean ± SD				
eGFR (Cockcroft), mL/min	92 ± 35	91 ± 32	82 ± 34	<0.001
Total cholesterol, mmol/L	191 ± 50	190 ± 46	186 ± 47	0.122
HDL cholesterol, mmol/L	52 ± 15	50 ± 14	46 ± 13	<0.001
LDL cholesterol, mmol/L	117 ± 42	117 ± 40	115 ± 40	0.404
Medication at discharge, No. (%)				
Aspirin	656 (96.2)	643 (95.5)	575 (94.6)	0.377
Adenosine diphosphate inhibitors	616 (90.3)	597 (88.7)	536 (88.2)	0.425
Ca antagonist	112 (16.4)	115 (17.1)	124 (20.4)	0.143
Beta blockers	561 (82.3)	562 (83.5)	527 (86.7)	0.086
ACE inhibitors	420 (61.6)	386 (57.4)	387 (63.7)	0.061
Angiotensin receptor blockers	103 (15.1)	128 (19.0)	138 (22.7)	0.002
Allopurinol	70 (10.2)	135 (20)	157 (25.8)	0.003
Statins	640 (93.8)	629 (93.5)	566 (93.1)	0.862

Values are given as mean and standard deviation or numbers and percentages. CABG = coronary artery bypass grafting, CAD = coronary artery disease, eGFR = estimated glomerular filtration rate, HDL = high-density lipoprotein, LDL = low-density lipoprotein, LVEF = left ventricular ejection fraction, PCI = percutaneous coronary intervention, ACE inhibitors = angiotensin convertase enzyme inhibitors.

**Table 2:** Angiographic and procedural characteristics stratified according to uric acid tertiles.

Characteristics	Tertile 1 <5.5 mg/dL (*n* = 682)	Tertile 2 5.6–6.9 mg/dL (*n* = 673)	Tertile 3 >7.0 mg/dL (*n* = 608)	*p*-Value
Lesion characteristics				
CTO target vessel				<0.001
LM	6 (0.9)	3 (0.4)	6 (1.0)	
LAD	217 (31.8)	186 (27.6)	140 (23.0)	
LCX	158 (23.2)	195 (29.0)	140 (23.0)	
RCA	301 (44.1)	289 (42.9)	322 (53.0)	
Vessel disease				0.098
1	140 (20.5)	119 (17.7)	99 (16.3)	
2	194 (28.4)	205 (30.5)	161 (26.5)	
3	348 (51.0)	349 (51.9)	348 (57.2)	
Multivessel disease	542 (79.5)	554 (82.3)	509 (83.7)	0.129
CTO length > 20 mm	499 (73.2)	511 (75.9)	483 (79.4)	0.031
Moderate/severe calcifications	372 (54.5)	371 (55.1)	355 (58.4)	0.333
Procedural characteristics				
Procedural success	573 (84)	559 (83.1)	498 (81.9)	0.602
Retrograde approach	150 (22.0)	146 (21.7)	175 (28.8)	0.004
Number of stents	1.28 ± 0.9	1.3 ± 0.9	1.3 ± 1.0	0.312
Total stent length, mm	33.7 ± 25.0	34.6 ± 26.2	36.8 ± 28.0	0.100
Contrast volume, mL	309 ± 151	326 ± 162	324 ± 153	0.083
Fluoroscopy time, min	33.8 ± 30.0	36.9 ± 47.0	38.5 ± 31.0	0.063
Kerma area product, cGy*cm^2^	11,047 ± 11,289	12,367 ± 13,307	14,220 ± 15,408	<0.001

Values are given as numbers or numbers and percentages of patients. CTO = chronic total occlusion, LAD = left anterior descending coronary artery, LCX = left circumflex coronary artery, LM = left main coronary artery, RCA = right coronary artery.

**Table 3:** Correlates of long-term mortality in the multivariable cox proportional hazards models.

Characteristics	HR	95% CI	*p*-Value
Sex	1.176	0.848–1.630	0.331
BMI	1.016	0.982–1.050	0.364
Diabetes mellitus	**1.349**	**1.031–1.766**	**0.029**
Arterial hypertension	**1.561**	**1.028–2.372**	**0.037**
Prior MI	1.116	0.842–1.478	0.446
LVEF > 40%	**0.441**	**0.335–0.579**	**0.000**
Renal function (Cockcroft clearance)	**0.977**	**0.972–0.981**	**0.000**
High-density lipoprotein [9]	0.999	0.990–1.009	0.897
CTO of RCA	3.349	0.459–24.441	0.233
CTO of RIA	3.265	0.445–23.956	0.245
CTO of LCX	3.851	0.526–28.204	0.184
Length of stent > 20 mm	0.945	0.704–1.268	0.706
Direction of PCI procedure (normal vs. contralateral or retrograde)	0.858	0.605–1.216	0.389
Uric acid Tertile 1 vs. Tertile 2	0.957	0.707–1.295	0.776
Uric acid Tertile 1 vs. Tertile 3	**0.671**	**0.491–0.916**	**0.012**
Successful CTO-PCI	**0.616**	**0.469–0.810**	**0.001**

HR = hazard ratio, CI = confidence interval, BMI = body mass index, MI = myocardial infarction, HDL = high-density lipoprotein, CTO = chronic total occlusion, RCA (ACD) = right coronary artery, RIA = ramus interventricularis anterior, LCX = left circumflex coronary artery, PCI = percutaneous coronary intervention, bold = statistically significant with *p* < 0.05.

## Data Availability

The datasets used and/or analyzed during the current study are available from the corresponding author upon reasonable request.

## References

[1] Cassar A., Holmes D.R., Rihal C.S., Gersh B.J. (2009). Chronic coronary artery disease: Diagnosis and management. Mayo Clin. Proc..

[2] Fefer P., Knudtson M.L., Cheema A.N., Galbraith P.D., Osherov A.B., Yalonetsky S., Gannot S., Samuel M., Weisbrod M., Bierstone D. (2012). Current perspectives on coronary chronic total occlusions: The Canadian Multicenter Chronic Total Occlusions Registry. J. Am. Coll. Cardiol..

[3] Stone G.W., Reifart N.J., Moussa I., Hoye A., Cox D.A., Colombo A., Baim D.S., Teirstein P.S., Strauss B.H., Selmon M. (2005). Percutaneous recanalization of chronically occluded coronary arteries: A consensus document: Part II. Circulation.

[4] Sianos G., Werner G.S., Galassi A.R., Papafaklis M.I., Escaned J., Hildick-Smith D., Christiansen E.H., Gershlick A., Carlino M., Karlas A. (2012). Recanalisation of chronic total coronary occlusions: 2012 consensus document from the EuroCTO club. EuroIntervention.

[5] Karmpaliotis D., Karatasakis A., Alasswad K., Jaffer F.A., Yeh R.W., Wyman R.M., Lombardi W.L., Grantham J.A., Kandzari D.E., Lembo N.J. (2016). Outcomes With the Use of the Retrograde Approach for Coronary Chronic Total Occlusion Interventions in a Contemporary Multicenter US Registry. Circ. Cardiovasc. Interv..

[6] Kearney K., Hira R.S., Riley R.F., Kalyanasundaram A., Lombardi W.L. (2017). Update on the Management of Chronic Total Occlusions in Coronary Artery Disease. Curr. Atheroscler. Rep..

[7] Sharaf El Din U.A.A., Salem M.M., Abdulazim D.O. (2017). Uric acid in the pathogenesis of metabolic, renal, and cardiovascular diseases: A review. J. Adv. Res..

[8] Ndrepepa G. (2018). Uric acid and cardiovascular disease. Clin. Chim. Acta Int. J. Clin. Chem..

[9] Tardif J.-C., Gregoire J., L’Allier P.L., Ibrahim R., Lesperance J., Heinonen T.M., Kouz S., Berry C., Basser R., Lavoie M.-A. (2007). Effects of Reconstituted High-Density Lipoprotein Infusions on Coronary Atherosclerosis: A Randomized Controlled Trial. JAMA.

[10] Tscharre M., Herman R., Rohla M., Hauser C., Farhan S., Freynhofer M.K., Huber K., Weiss T.W. (2018). Uric acid is associated with long-term adverse cardiovascular outcomes in patients with acute coronary syndrome undergoing percutaneous coronary intervention. Atherosclerosis.

[11] Ndrepepa G., Braun S., Haase H.U., Schulz S., Ranftl S., Hadamitzky M., Mehilli J., Schomig A., Kastrati A. (2012). Prognostic value of uric acid in patients with acute coronary syndromes. Am. J. Cardiol..

[12] Ndrepepa G., Braun S., King L., Hadamitzky M., Haase H.U., Birkmeier K.A., Schomig A., Kastrati A. (2012). Association of uric acid with mortality in patients with stable coronary artery disease. Metabolism Clin. Exp..

[13] Bickel C., Rupprecht H.J., Blankenberg S., Rippin G., Hafner G., Daunhauer A., Hofmann K.P., Meyer J. (2002). Serum uric acid as an independent predictor of mortality in patients with angiographically proven coronary artery disease. Am. J. Cardiol..

[14] Guerra A., Rangan B.V., Coleman A., Xu H., Kotsia A., Christopoulos G., Sosa A., Chao H., Han H., Abdurrahim G. (2015). Effect of Extended-Release Niacin on Carotid Intima Media Thickness, Reactive Hyperemia, and Endothelial Progenitor Cell Mobilization: Insights From the Atherosclerosis Lesion Progression Intervention Using Niacin Extended Release in Saphenous Vein Grafts (ALPINE-SVG) Pilot Trial. J. Invasive Cardiol..

[15] Del Pinto R., Viazzi F., Pontremoli R., Ferri C., Carubbi F., Russo E. (2021). The URRAH study. Panminerva Med..

[16] Borghi C., Piani F. (2021). Uric Acid and Risk of Cardiovascular Disease: A Question of Start and Finish. Hypertension.

[17] Patetsios P., Song M., Shutze W.P., Pappas C., Rodino W., Ramirez J.A., Panetta T.F. (2001). Identification of uric acid and xanthine oxidase in atherosclerotic plaque. Am. J. Cardiol..

[18] Yu M.A., Sanchez-Lozada L.G., Johnson R.J., Kang D.H. (2010). Oxidative stress with an activation of the renin-angiotensin system in human vascular endothelial cells as a novel mechanism of uric acid-induced endothelial dysfunction. J. Hypertens..

[19] Forstermann U., Xia N., Li H. (2017). Roles of Vascular Oxidative Stress and Nitric Oxide in the Pathogenesis of Atherosclerosis. Circ. Res..

[20] Spiga R., Marini M.A., Mancuso E., Di Fatta C., Fuoco A., Perticone F., Andreozzi F., Mannino G.C., Sesti G. (2017). Uric Acid Is Associated With Inflammatory Biomarkers and Induces Inflammation Via Activating the NF-kappaB Signaling Pathway in HepG2 Cells. Arter. Thromb. Vasc. Biol..

[21] Mehran R., Claessen B.E., Godino C., Dangas G.D., Obunai K., Kanwal S., Carlino M., Henriques J.P., Di Mario C., Kim Y.H. (2011). Long-term outcome of percutaneous coronary intervention for chronic total occlusions. JACC Cardiovasc. Interv..

[22] Kanbay M., Segal M., Afsar B., Kang D.H., Rodriguez-Iturbe B., Johnson R.J. (2013). The role of uric acid in the pathogenesis of human cardiovascular disease. Heart.

[23] Borghi C., Agnoletti D., Cicero A.F.G., Lurbe E., Virdis A. (2022). Uric Acid and Hypertension: A Review of Evidence and Future Perspectives for the Management of Cardiovascular Risk. Hypertension.

[24] Noma K., Kihara Y., Higashi Y. (2017). Is Serum Uric Acid a Biomarker, but not a Mediator in Patients With Lifestyle and Cardiovascular Diseases?. Int. Heart J..

[25] Kao M.P., Ang D.S., Gandy S.J., Nadir M.A., Houston J.G., Lang C.C., Struthers A.D. (2011). Allopurinol benefits left ventricular mass and endothelial dysfunction in chronic kidney disease. J. Am. Soc. Nephrol. JASN.

[26] Lee P.H., Lee S.W., Park H.S., Kang S.H., Bae B.J., Chang M., Roh J.H., Yoon S.H., Ahn J.M., Park D.W. (2016). Successful Recanalization of Native Coronary Chronic Total Occlusion Is Not Associated With Improved Long-Term Survival. JACC. Cardiovasc. Interv..

[27] Kirschbaum S.W., Baks T., van den Ent M., Sianos G., Krestin G.P., Serruys P.W., de Feyter P.J., van Geuns R.J. (2008). Evaluation of left ventricular function three years after percutaneous recanalization of chronic total coronary occlusions. Am. J. Cardiol..

[28] Lee K.K., Doudesis D., Anwar M., Astengo F., Chenevier-Gobeaux C., Claessens Y.E., Wussler D., Kozhuharov N., Strebel I., Sabti Z. (2022). Development and validation of a decision support tool for the diagnosis of acute heart failure: Systematic review, meta-analysis, and modelling study. BMJ.

[29] Behnes M., Brueckmann M., Lang S., Espeter F., Weiss C., Neumaier M., Ahmad-Nejad P., Borggrefe M., Hoffmann U. (2013). Diagnostic and prognostic value of osteopontin in patients with acute congestive heart failure. Eur. J. Heart Fail..

[30] Behnes M., Bertsch T., Weiss C., Ahmad-Nejad P., Akin I., Fastner C., El-Battrawy I., Lang S., Neumaier M., Borggrefe M. (2016). Triple head-to-head comparison of fibrotic biomarkers galectin-3, osteopontin and gremlin-1 for long-term prognosis in suspected and proven acute heart failure patients. Int. J. Cardiol..

[31] Goliasch G., Winter M.P., Ayoub M., Bartko P.E., Gebhard C., Mashayekhi K., Ferenc M., Buettner H.J., Hengstenberg C., Neumann F.J. (2019). A Contemporary Definition of Periprocedural Myocardial Injury After Percutaneous Coronary Intervention of Chronic Total Occlusions. JACC Cardiovasc. Interv..

[32] Kim S.H., Behnes M., Mashayekhi K., Bufe A., Meyer-Gessner M., El-Battrawy I., Akin I. (2021). Prognostic Impact of Percutaneous Coronary Intervention of Chronic Total Occlusion in Acute and Periprocedural Myocardial Infarction. J. Clin. Med..

